# ADAMTS5 Orchestrates Cell Lineage Specific Patterning and Extracellular Matrix Organization During Semilunar Valve Development

**DOI:** 10.3390/jcdd12090371

**Published:** 2025-09-19

**Authors:** Loren E. Dupuis, Joshua J. Mifflin, Amy L. Marston, Jeremy P. Laxner, Christine B. Kern

**Affiliations:** Department of Regenerative Medicine and Cell Biology, Medical University of South Carolina, Charleston, SC 29425, USA

**Keywords:** aortic valve, semilunar valves, versican, ADAMTS5, cardiac neural crest, extracellular matrix, proteoglycan, EndoMT, valvular interstitial cells

## Abstract

Aortic valve (AV) disease affects about 5% of the aging population, with AV replacement as the only treatment option. Histopathology indicates that accumulation of extracellular matrix (ECM) proteoglycans correlates with dysfunctional AVs. Proteoglycan content is controlled by ECM proteolytic cleavage, with the cleaved and intact forms of the proteoglycan Versican (VCAN) occupying different cell lineage-specific regions throughout AV development. To test the hypothesis that VCAN cleavage is required for lineage specific cell behaviors and ECM stratification, the cardiac neural crest (CNC) lineage was traced in mice with global inactivation of the proteoglycan protease *Adamts5*. By mid-gestation, *Adamts5^−/−^* mice exhibited disorganized CNC patterning with excess VCAN and enlarged semilunar valve (SLV) morphology. Use of the *Adamts5* floxed mice indicated that *Adamts5* was required in the endothelial cells and their mesenchymal derivatives (EndoMT lineage) to prevent VCAN accumulation, initiate ECM stratification, and promote normal SLV morphology. These data suggest that the ECM remodeling event of VCAN cleavage may orchestrate cell lineage distinct behaviors and interactions to control proteoglycan levels throughout AV development and to prevent disease. Understanding mechanisms that regulate VCAN content may lead to the discovery of effective pharmacological targets for the treatment of AV disease.

## 1. Introduction

Aortic valve disease, particularly aortic stenosis, is a common condition affecting about 5% of people aged 65 and older. Changes in aortic valve (AV) morphology coincide with the onset of symptoms including aortic root dilation, aortic regurgitation, and narrowing of the AV [1]. Patients with a bicuspid aortic valve (BAV), that has two rather than three cusps, can have earlier onset and more severe AV disease. BAVs are also an independent risk factor for ascending aortic artery aneurisms, which can rupture and result in sudden death. Advances in imaging techniques have led to increased detection rates of congenital heart defects (CHD), particularly for milder AV malformations, but it is unclear if subtle AV changes that occur during development predispose patients to AV disease later in life. Treatments for AV disease are limited to AV replacement surgery or transcatheter AV implantation, since there are no effective medications for AV disease.

Healthy cardiac valves exhibit normal morphology with a highly specialized extracellular matrix (ECM). Histopathology of diseased AVs reveals disorganization and expansion of the ECM, with massive excesses of aggregating proteoglycans Versican (VCAN) and Aggrecan (ACAN) [2,3,4,5,6]. In murine models of AV disease, excess proteoglycans are also associated with abnormal AV morphology and ascending aortic artery anomalies (aortopathies) [7,8]. Normally, proteoglycan content is controlled by ECM proteases that cleave proteoglycans for clearance from the ECM. Since excess proteoglycans have emerged as a potential hallmark of cardiac valve and vessel diseases, investigating mechanisms that control proteoglycan content may lead to the discovery of effective pharmaceutical agents to treat and prevent AV disease.

In this study we utilized a developmental approach to investigate mechanisms that are dependent on VCAN cleavage during valve formation. The developmental precursor of AVs and pulmonary valves (PV), collectively the semilunar valves (SLVs), as well as the ascending aortic and pulmonary arteries, is the cardiac outflow tract (OFT). In early heart formation, the OFT resembles a simple tube, with an outer muscular sleeve [9,10,11,12,13] and an inner layer of endothelial cells. OFT cushions develop in between the myocardial and endothelial layers by deposition of ECM components VCAN and hyaluronan (HA) (often referred to as cardiac jelly) [14]. The OFT cushions regulate blood flow in the early embryo and are populated with several different cell lineages, including the cardiac neural crest (CNC). CNC originates from the hindbrain, migrates throughout the OFT, and represents the majority of mesenchymal cells in the early OFT cushions. CNC condenses and facilitates fusion of opposing cushions, which divides the common OFT into the pulmonic and aortic channels [15,16,17]. CNC also contributes to the valvular interstitial cells (VICs) in the PV and AV cusps and gives rise to smooth muscle cells of the ascending aorta [12,18]. Mesenchymal cells generated from an epithelial to mesenchymal transition (EndoMT) also populate the OFT; this lineage occupies a portion of the proximal cushions and contributes to VICs of the semilunar valves [19,20]. A subset of secondary heart field (SHF) myocardial cells (Tnnt2-Cre+) contributes to the anterior cusp (An) of the PV and the non-coronary (NC) cusp of the AV [21,22]. Although the patterning of CNC, EndoMT, and Tnnt2-Cre cell lineages are reproducible, factors that orchestrate lineage specific behaviors and cell–cell interactions during development are not well understood.

Throughout OFT development, there is a dramatic remodeling of the ECM, including proteolytic breakdown of VCAN. [23,24]. Mice deficient in the ECM protease A Disintegrin and Metalloproteinase with ThromboSpondin motifs 5 (ADAMTS5), which cleaves VCAN, exhibit enlarged SLV and aortopathies, with massive excesses of VCAN and Acan, respectively [8,25]. Since in vivo reduction in VCAN rescues the enlarged semilunar valve phenotype, the mechanism of VCAN cleavage appears essential for OFT development [25]. Here, ADAMTS5-deficient mice revealed that CNC patterning was disrupted in early OFT development, consistent with a loss in migration and cell condensation in regions where cleaved VCAN fragments normally reside. Tie2-Cre deletion of *Adamts5* indicated that the EndoMT expression of *Adamts5* was required to restrict VCAN and promote cell condensation in the SLV cusps. Therefore, the ECM remodeling event of VCAN cleavage may be a key factor that coordinates cell behavior and mechanisms critical for normal AV development and to prevent AV disease.

## 2. Materials and Methods

### 2.1. Gene-Targeted Mice

The housing and care of mice and all the procedures used in these studies were performed in accordance with the ethical guidelines and regulations that were approved by the Medical University of South Carolina Institutional Animal Care and Use Committee (# AUP-25-64) on 29 July 2025. Mice were housed in individually ventilated cages (IVCs) within an HEPA-filtered room maintained at 22 °C and 50% humidity, with a 12 h light/dark cycle. Cages contained autoclaved corn cob bedding, nesting material, and a food hopper with standard rodent chow. Water was provided ad libitum via bottles fitted with sipper tubes. Mice were group-housed with littermates, and all husbandry procedures followed institutional guidelines to minimize stress and ensure animal welfare. Tie2-Cre, (B6.Cg-Tg(Tek-cre)1Ywa/J); Wnt1-Cre, (129S4.Cg-Tg(Wnt1-Cre)2Sor/J); Tnnt2-Cre, (Tg(Tnnt2-cre)5Blh/JiaoJ); tdTomato-(Enhanced Green Fluorescent Protein (EGFP) reporter, (B6.129(Cg)-Gt(ROSA)26Sor^tm4(ACTB-tdTomato,-EGFP)Luo^/J) were purchased from Jackson laboratories. The backgrounds of mice that contained a combination of Cre and Rosa transgenes were mixed; 129 strain backcross to C57/BL6 for 6-8 generations. This study utilized mice containing a global knockout of the ECM proteoglycan protease ADAMTS5 B6.129P2-*Adamts5tm1Dgen*/J (referred to as *Adamts5^−/−^*) [25]; these mice exhibit enlarged, malformed SLV (PV and AVs), as well as, anomalies of the ascending aortas (aortopathies). There were no double outlet right ventricles or persistent truncus arteriosus defects observed in the *Adamts5^−/−^* hearts. *Adamts5^−/−^* malformations correlate with excess proteoglycans VCAN and ACAN, respectively [8,25].

To perform lineage tracing, the Wnt1-Cre, Tie2-Cre or Tnnt2-Cre transgene was used in combination with the tdTomato-EGFP reporter in the *Adamts5^−/−^* or control, *Adamts5^+/+^*. With this strategy, the tdTomato gene was excised in the Cre positive cells, to allow expression of EGFP (Supplemental Figure S1). To generate each Cre lineage tracing, a male mouse containing the Cre of interest (Tie2, Wnt1 or Tnnt2) was mated to a female containing two copies of the TdTomato-EGFP reporter. The embryos or hearts were harvested from the mating and mice generated from this mating were not used for breeding in other strategies, thus keeping the Cre in the male germ line. Littermates of *Adamts5^−/−^* and *Adamts5^+/+^* were used in all studies to minimize experimental variability.

To determine the requirement of *Adamts5* mRNA expression in a specific lineage we utilized the *Adamts5* floxed allele designated (*Adamts5^f/f^*) [26,27]. In combination with a Cre transgene, the *Adamts5^fl/fl^* removes exon 3 that encodes the proteoglycan cleavage domain (Supplemental Figure S2). These mice were used to investigate the contribution of *Adamts5* from Tie2-Cre or Tnnt2-Cre OFT lineages during development.

### 2.2. Histology, Immunohistochemistry and In Situ Hybridization

Embryos or isolated hearts were fixed in either 4% paraformaldehyde or the perceptive fixative (35% methanol; 35% acetone; 5% acetic acid; 25% water) and mounted in paraffin blocks. Sections of 5 μm in length were generated and used for immunohistochemistry (IHC) and H&E. Primary antibodies generated against VCAN MilliporeSigma AB1033, Rabbit anti-Mouse VCAN Gagβ, cleaved VCAN (DPEAAE) ThermoFisher PA1-1748A, Waltham MA, USA, α smooth muscle actin (Sigma, A5228 Cambridge, MA, USA), and α sarcomeric actin (Sigma, A2172 Cambridge, MA, USA) were used as previously published [25,28]. To detect EGFP, the anti-EGFP antibody (abcam ab13970) and secondary anti-chick-IgY Alexa Fluor^®^ 647 (Jackson ImmunoResearch, West Grove, PA, USA) were utilized since the anti-EGFP antibody yielded a more robust signal than the direct florescence of EGFP in the heart sections. In addition, Cre;EGFP heart images were generated using the Leica SP8 to view fluorescence from Cre negative cells (TdTomato) and Cre recombined lineage traced cells (EGFP). To ensure cells that were negative for EGFP were positive for Tdtomato, indicating a lack of Cre recombination, the TdTomato fluorescence was performed for each Cre lineage.

### 2.3. Three- Dimensional Reconstructions

Three-dimensional (3D) reconstructions were generated using Amira™ 5.3.3 (Visage Imaging, Andover, MA, USA) [25]. For 3D images of lineage contribution, IHC was performed using an anti-EGFP (Ab13970, Abcam, Cambridge, UK) on each section for EGFP lineage tracing. EGFP antibodies were used in combination with VCAN and a muscle marker, either α smooth muscle actin (SMA) (E12.5) or α-sarcomeric actin (Sarc) (E11.5) (with or without propidium iodide (PI)). For reconstructions, label fields were generated using thresholding from the EGFP positive Cre-expressing cells in each section. The thresholding generated a label field that was then colorized to mark the positive pixels. Each subsequent image was then stacked on the previous image in Amira™ to generate the 3D reconstruction of EGFP positive cells within the outflow tract or portion of the valve cusps, as indicated. The volume (in voxels) of positive cells was generated using Amira™. To generate 3D reconstructions for valve morphology and volume determination approximately 90, 5 μm-thick paraffin sections were used for E11.5 whole hearts and approximately 50 sections for E14.5 and E17.5 PV and AV. Label fields were generated for each cusp and their corresponding hinge region of the PV or AV.

At E12.5, the valve cusp mesenchyme was defined from the point at which the major cushions fuse and the unfused margins stained positive for VCAN. For Tie2-Cre;EGFP experiments, the endothelial cells were excluded from the 3D reconstructions, i.e., only Tie2-Cre positive mesenchymal cells were utilized. For the Tnnt2-Cre quantification, EGFP within the cusp mesenchyme, but not the outer myocardial sleeve, was used.

### 2.4. OFT CNC Lineage Contribution

The percentage of CNC lineage cells throughout the OFT at E11.5 was determined by counting the number of EGFP positive cells compared to the total number of cells in the cushion mesenchyme. Beginning at the most distal point of the OFT cushions, four sections per OFT were used from each mouse and averaged in 20 μm increments throughout the entire OFT. Data used in this manuscript represents n = 6 OFTs of *Adamts5^+/+^* and n = 7 *Adamts5^−/−^*.

### 2.5. Statistics

The power calculation yielded a sample size of 3 per group, and an adjustment to n = 5 was made to the sample size to account for phenotypic changes between the *Adamts5^−/−^* and *Adamts5^+/+^* SLV. Statistical analyses were performed by using Student’s t-test for two group comparisons and two-way analysis of variance (ANOVA) for multiple comparisons. The F-test was used to compare variances between groups. If the variances were significant, the non-parametric Wilcoxon test was used. To control for slight developmental differences and genetic backgrounds of the Cre lineage strains, cell numbers were expressed as a percentage of the *Adamts5^+/+^*. Each histomorphometry and IHC analysis was performed in an experimental replicate of at least 3 to provide estimates of variance. An alpha level of <0.05 was considered significant. For ex vivo assays, a sample size of 4 per condition were utilized, yielding sufficient power (92%) to detect a difference in mean cell count. Pertinent statistical information is provided in the figure legends. Each symbol on the graph represents the data point from one mouse. Investigators used number randomization and were not aware of genotype grouping prior to data analysis. Data are represented on graphs with bars representing the mean, and no data points were excluded. Statistical analyses were generated using GraphPad Prism version 9.0 for Mac, GraphPad Software, San Diego, CA, USA.

## 3. Results

### 3.1. CNC Were Reduced in the Proximal OFT of Adamts5^−/−^ Hearts with Excess VCAN

**Figure 1:**
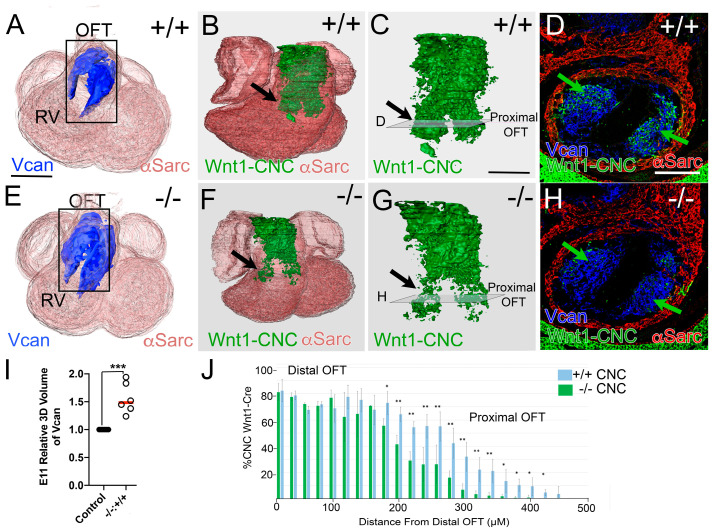
Wnt1-Cre cardiac neural crest (CNC) lineage cells were reduced in the proximal OFT in *Adamts5^−/−^* hearts at embryonic day 11.5 (E11.5). Three-dimensional (3D) immunohistochemical (IHC) reconstructions of Versican (VCAN; blue), Wnt1-Cre (CNC; green), and αSarcomeric actin (αSarc, red) of whole hearts from *Adamts5^+/+^* (**A**–**C**; +/+) and *Adamts5^−/−^* (**E**–**G**; −/−) mice. Black rectangle in (**A**,**E**) designate outflow tract (OFT). Black arrows (**B**,**C**,**F**,**G**)—regions in the proximal OFT with Wnt1-Cre positive cells. Green arrows (**D**,**H**) indicate CNC in proximal sections reduced in the *Adamts5^−/−^* hearts (**H**). Transparent squares (designated **D**,**H**; in panels **C**,**G**) indicate location of 2D sections in (**D**) and (**H**), respectively. Green arrows (**D**,**H**) show Wnt1-Cre positive cells in the proximal OFT, reduced in *Adamts5^−/−^* OFTs (**H**). Graph in (**I**)-relative VCAN volumes in E11 hearts from closed circles—*Adamts5^+/+^*; open circles—*Adamts5^−/−^*; parametric test. Graph in (**J**)—percentage of Wnt1-Cre positive CNC in sections throughout the OFT non-parametric test; 0—defines the distal most region of the OFT (blue bars—*Adamts5^+/+^*, −/−, n = 6; green bars—*Adamts5^−/−^*, −/−, n = 7). * *p* < 0.05, ** *p* < 0.01 *** *p* < 0.001. Bar in **A** = 150 μm, **B** = 50 μm.

The first set of experiments in this study utilized mice containing a global knockout of the ECM proteoglycan protease ADAMTS5, referred to as *Adamts5^−/−^* [25]; these mice exhibit enlarged, malformed semilunar valves (PV and AVs) with 100% penetrance, as well as aortopathies. *Adamts5^−/−^* SLV malformations correlate with excess VCAN [8,25]. Three-dimensional reconstructions of VCAN localization in the E11.5 OFTs revealed a consistent increase in VCAN in *Adamts5^−/−^* compared to *Adamts5^+/+^* OFTs (Figure 1A,E,F). To determine the consequence of excess VCAN to the CNC lineage, Wnt1-Cre transgene in combination with the tdTomato-EGFP reporter (tdT-EGFP) was used in both the *Adamts5^+/+^* and *Adamts5^−/−^* mice. Three-dimensional reconstructions of the CNC lineage tracing in E11.5 whole hearts were generated by immunolocalization of EGFP (CNC lineage), αSMA, and VCAN in each histological section. In *Adamts5^+/+^* E11.5 OFTs, the CNC had migrated and colonized cushions throughout the OFT (Figure 1B–D, green). However, in the *Adamts5^−/−^* OFT, there appeared to be less CNC in the proximal region (Figure 1F–H black arrows, green). Sections from the proximal OFT of *Adamts5^+/+^* (D, green arrows, cells) and *Adamts5^−/−^* also showed a reduced number of CNC lineage in the *Adamts5^−/−^* OFTs (Figure 1H, green arrows, cells). Assessment of the Wnt1-Cre positive CNC throughout the OFT demonstrated a significant reduction in CNC in the *Adamts5^−/−^* proximal OFT (n = 7) compared to *Adamts5^+/+^* littermates (n = 6; Figure 1J; *Adamts5^+/+^* (+/+) blue bars, *Adamts5^−/−^* (−/−) green bars).

### 3.2. Reduction in CNC in the Adamts5^−/−^ Proximal OFT Correlated with an Increase in the EndoMT Lineage

**Figure 2:**
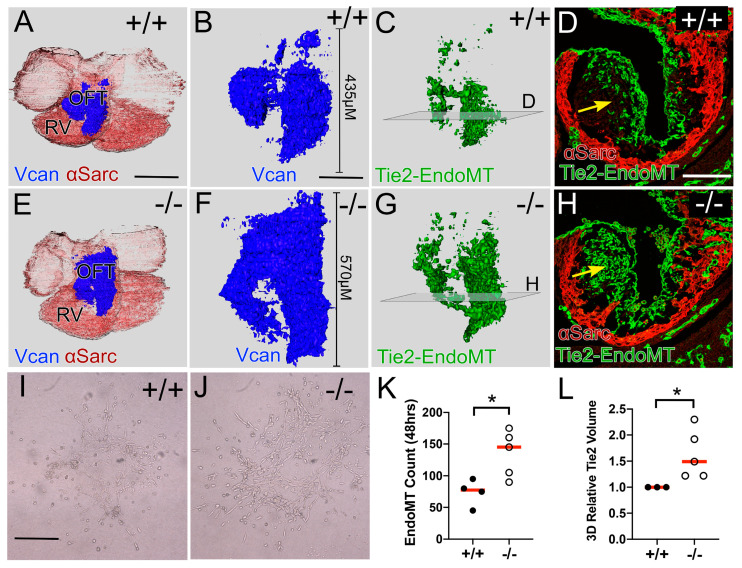
Tie2-Cre, endothelial to mesenchymal transition (EndoMT) lineage cells were increased in the proximal OFT of *Adamts5^−/−^*. Whole hearts at E11.5 of *Adamts5^+/+^* (**A**–**C**; +/+) and *Adamts5^−/−^* (**E**–**G**; −/−) were reconstructed from sections immunostained for αSarc (red); VCAN (blue) and the Tie-2-Cre lineage (green). Transparent squares in (**C**,**G**) proximal OFTs correspond to the sections shown in (**D**) and (**H**), respectively. Increase in Tie2-EGFP derived cells in *Adamts5^−/−^* proximal OFT section (**H**, green, yellow arrow) compared to *Adamts5^+/+^* OFT (**D**; yellow arrow). EndoMT Assay (**I**–**K**) from *Adamts5^+/+^* (**I**) and *Adamts5^−/−^* (**J**) explants graphed in **K**, parametric test. Graph in **L** shows quantification of the Tie2-Cre lineage volume in E11.5 hearts, non-parametric test; each symbol represents a single OFT explant. Closed circles (**K**,**L**) *Adamts5^+/+^* and open circles (**K**,**L**) *Adamts5^−/−^*. Red bars (**K**,**L**)—standard deviation. Bar in (**A**) = 150 μm, (**B**) = 100 μm, (**I**) = 50 μm. Graph in (**L**), relative change in 3D Recons of Tie2-Cre lineage of *Adamts5^−/−^* (* *p* < 0.05). Red bars (**K**,**L**) = mean. Bar in (**A**) = 150 μm, (**B**) = 50 μm.

The EndoMT lineage, which consists of the endothelial cells as well as their mesenchymal derivatives, was mapped in *Adamts5^+/+^* (Figure 2C,D) and *Adamts5^−/−^* OFTs (Figure 2G,H) using the Tie2-Cre promoter and the tdTomato-EGFP reporter. The increase in Vcan observed in the *Adamts5^−/−^* OFT (Figure 2A,B,E,F) correlated with the increase in Tie2-Cre lineage in the proximal OFT of *Adamts5^−/−^* (Figure 2G,H; green). The ex vivo EndoMT assay, that quantifies the endothelial to mesenchymal cell transitions, revealed an increase in mesenchymal cells from the endothelial layer of *Adamts5^−/−^* explants compared to *Adamts5^+/+^* (Figure 2I,J,K graph). Loss of *Adamts5* resulted in an increase in the EndoMT transition, indicating *Adamts5* expression may be required to control EndoMT during early OFT remodeling.

### 3.3. The Prevalvular Complex of the Developing OFT Was Altered by Loss of VCAN Cleavage by E11.5

**Figure 3:**
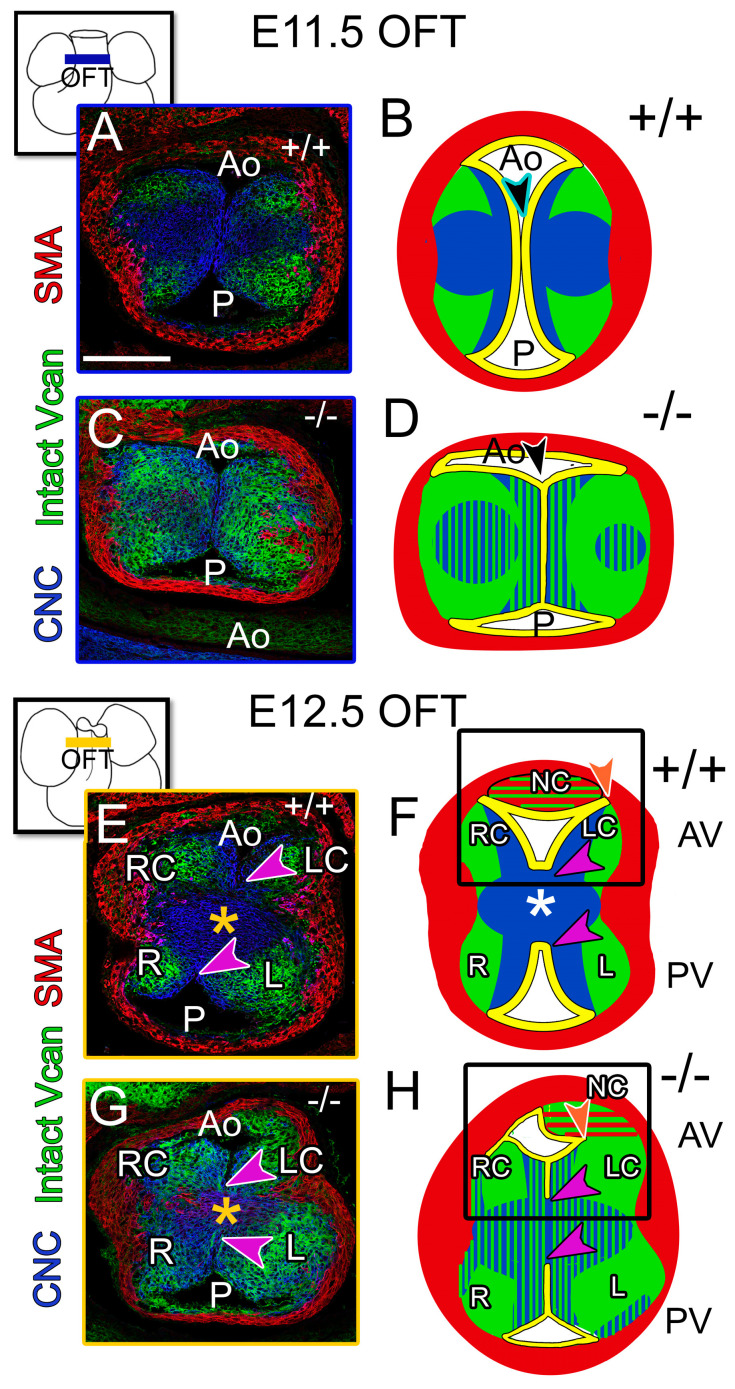
The prevalvular complex was disrupted in *Adamts5^−/−^* OFTs. A cross section of E11.5 OFTs (**A**–**D**): IHC (**A**,**C**); schematic (**B**,**D**); outset A, blue square. CNC (blue, **A**–**D**), VCAN (green, **A**–**D**) IHC of α-smooth muscle actin (red, myocardium) in *Adamts5^−/−^* (**C**,**D**). Unfused margins in **B** of *Adamts5^+/+^* (black arrowhead, aqua stroke) appear fused in *Adamts5^−/−^* (**D**, black arrowhead, white stroke). Excess VCAN (green, **C**,**D**) correlates with loss of CNC patterning in the *Adamts5^−/−^* prevalvular complex. Heart outline, (**E** outset orange square) denotes cross section of E12.5 medial OFTs of *Adamts5^+/+^* (**E**,**F**) and *Adamts5^−/−^* (**G**,**H**). *Adamts5^+/+^* AV cusps (**F**, black box outline): CNC (blue) reside near the endocardium (yellow), VCAN in the lateral region (green, **E**,**F**), myocardial lineage (red). *Adamts5^−/−^* fusion of the endothelium (**G**,**H**, purple arrowhead) and endothelium (yellow) has not invaginated to the myocardium in the *Adamts5^−/−^* (**H**, orange arrowhead) as in the *Adamts5^+/+^* (**F**, orange arrowhead). Bar in (**A**) = 100 μm and applies to (**B**–**F**).

The prevalvular complex is a model of OFT development that includes cushion tissue morphology, cell lineage patterning, VCAN -rich ECM, and myocardial localization [29]. (Note that αSMA labels the myocardial sleeve and is not yet specific for the arterial walls.) Comparison of the *Adamts5^−/−^* E11.5 prevalvular complex to *Adamts5^+/+^* identified several characteristics that may contribute to the semilunar valve defects in *Adamts5^−/−^* mice (n = 14). *Adamts5^−/−^* OFT cushions exhibited a block shaped appearance (Figure 3C,D) compared to more tapered cushions of E11.5 *Adamts5^+/+^* littermates (n = 8) (Figure 3A,B). In *Adamts5^+/+^* OFTs, VCAN and CNC occupy distinct regions of the prevalvular complex, but in *Adamts5^−/−^* OFTs (Figure 3A), VCAN and CNC were interspersed (Figure 3C,D blue/green). By E12.5 in the medial OFT, the major cushions have fused, generating the separate aortic (Ao) and pulmonary channels (P). Fusion of the cushions in the *Adamts5^−/−^* OFTs resulted in ectopically fused endothelium at the outer margines of the major cushions between the L and R cusps of the PV and the LC and RC cusps of the AV (Figure 3G,H, purple arrows compared to E,F, purple arrows). Based on these data, loss of *Adamts5* disrupted the prevalvular complex with excess VCAN and altered CNC patterning.

### 3.4. At E12.5, the CNC Patterning and Myocardial Lineage Contribution Were Disrupted in the Developing Valve Cusps in ADAMTS5-Deficient Distal OFTs

**Figure 4:**
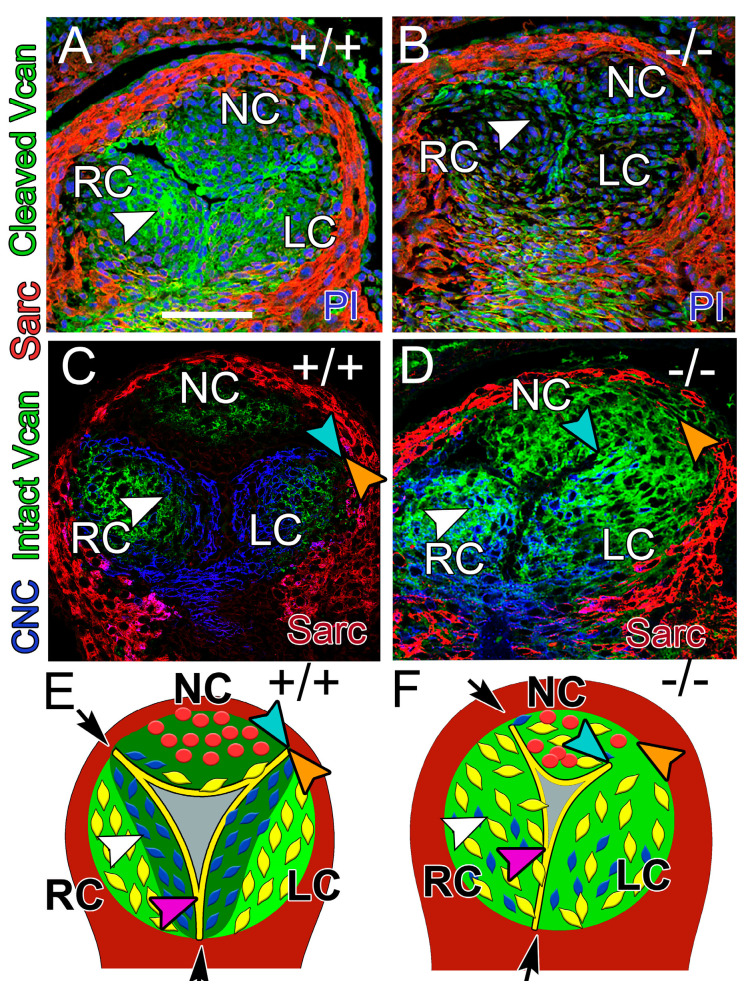
CNC patterning and endothelial invagination were disrupted in E12.5 *Adamts5^−/−^* AV cusps. *Adamts5^+/+^* (**A**,**C**,**E**; n = 9) and *Adamts5^−/−^* AV cusps (**B**,**D**,**F**; n = 10). Cleaved VCAN (**A**,**B**; green); αSarcomeric actin (red; **A**,**B**), propidium iodide (blue, PI; **A**,**B**); intact VCAN (green, **C**,**D**); CNC lineage (blue, **C**,**D**). Schematic of E12.5 AV cusps (**E**,**F**): yellow—EndoMT lineage, blue—CNC lineage, red—Tnnt2-Cre myocardial cells, cleaved VCAN (dark green, **E**); intact VCAN (bright green, **E**,**F**). White arrowheads (**A**,**C**,**E**) overlap of cleaved VCAN and CNC (blue, **C**,**E**) disrupted in *Adamts5^−/−^* (**B**,**D**,**F**). Gap in endothelial invagination (orange–blue arrowhead; **D**,**F**) compared to *Adamts5^+/+^* (orange–blue arrowheads; **C**,**E**). Purple arrowheads—endothelial fusion between the RC and LC. Bar in **A** = 50 μm.

In the distal E12.5 OFT, endothelium divides the cushion mesenchyme into the three separate AV cusps (Figure 4: RC—right coronary, NC—non-coronary, LC—left coronary) and is predominantly associated with cleaved VCAN (Figure 4A, white arrowhead, green), while intact VCAN is associated with the region of the cusps adjacent to the myocardium (Figure 4C, green). This pattern of VCAN localization is disrupted in the *Adamts5^−/−^* AV (Figure 4B,D, green). In *Adamts5^+/+^* prevalvular cusps, CNC clustered adjacent to the endothelium (Figure 4C, blue), where cleaved VCAN localized (Figure 4A, white arrowhead) and was void of intact VCAN (Figure 4C, bright green). In contrast, *Adamts5^−/−^* had undetectable levels of cleaved VCAN (Figure 4B, white arrowhead) and CNC did not compact in the valve cusp (Figure 4D, white arrowhead). The endothelial cells that divide cushion tissue into separate cusps were disrupted in *Adamts5^−/−^* (n = 7) compared to *Adamts5^+/+^* (n = 9) (Figure 4C–F, blue–orange arrows). The space between the endocardium (Figure 4C–F, blue arrows) and the myocardium (Figure 4C–F, orange arrows) was greater in *Adamts5^−/−^* (n = 7) prevalvular cusps compared to *Adamts5^+/+^* (n = 9). The distance between the blue and orange denotes a failure of the endocardium to invaginate and separate the cushion tissue into three separate cusps, which may lead to malformations including raphes and BAVs.

### 3.5. The Myocardial (Tnnt2-Cre) Population Was Reduced in the Adamts5^−/−^ PV

**Figure 5:**
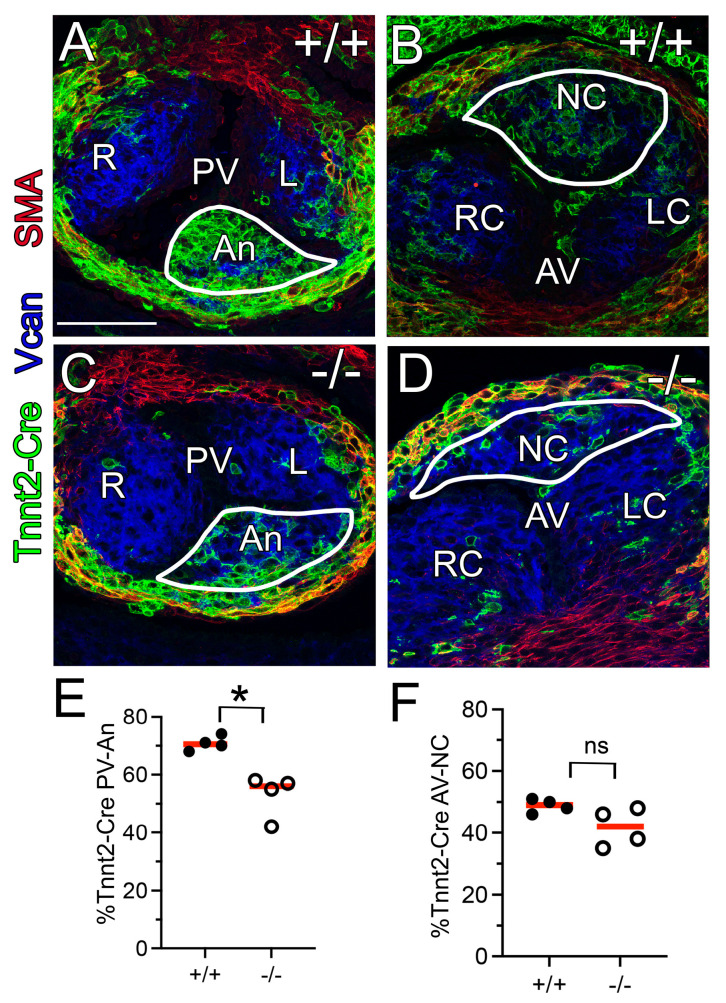
Tnnt2-Cre myocardial lineage was reduced in the An cusp of the PV. E12.5, Tnnt2-Cre lineage (**A**–**D**, green). PV-An of the *Adamts5^+/+^* (**A**, green, white outline) compared to PV-An *Adamts5^−/−^* (**C**, green, white outline). AV-NC cusp of the *Adamts5^+/+^* (**B**, green, outline white) compared to AV-NC An of the *Adamts5^−/−^* (**D**, green, outline white). VCAN (blue); αSMA (red). Note the overlap of the Tnnt2-Cre labeled myocardial sleeve with the αSMA at E12.5. Quantification of Tnnt2-Cre cells in the total depth of PV-An *Adamts5^+/+^* (**E**, closed circles, n = 4) and PV-NC of *Adamts5^−/−^* (**E**, open circles, n = 4, * non-parametric test, * *p* < 0.05). Quantification of the Tnnt2-Cre lineage of the AV-NC *Adamts5^+/+^* (**F**, closed circles, n = 4) and AV-NC of *Adamts5^−/−^* (**F**, open circles, n = 4), ns—not significant. Bar in (**A**) = 50 μm.

We and others have determined that the myocardial Tnnt2-Cre lineage populates most of the developing NC cusp of the AV and Anterior cusp (An) of the PV [21,22]. Myocardial lineage tracing using the Tnnt2-Cre and tdTomato-EGFP reporter in *Adamts5^+/+^* (Figure 5A,C) and *Adamts5*-deficient mice (Figure 5B,D) indicated that the Tnnt2-Cre lineage in the An cusp of the PV (E12.5; n = 4) was significantly reduced in the *Adamts5^−/−^* mice compared to *Adamts5^+/+^* PV (n = 4; Figure 5E, * *p* < 0.05). Although the reduction in the Tnnt2-Cre lineage in the *Adamts5^−/−^* NC of the AVs was not statistically significant, it may have biological consequences in developing AV in vivo (Figure 5F).

### 3.6. Adamts5 mRNA, Expressed by the Endothelium and Myocardium, Were Required for Normal Semilunar Valve Formation

#### 3.6.1. ADAMTS5 Was Required by the EndoMT Lineage for Normal SLV Development

**Figure 6:**
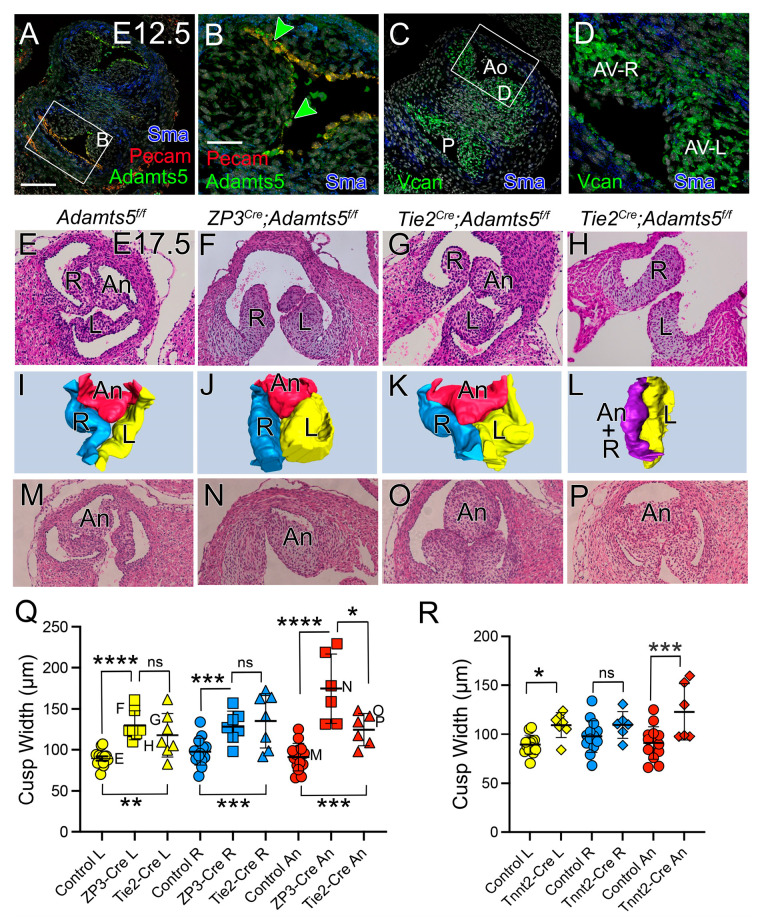
*Adamts5* expression in the Tie2-Cre and Tnnt2-Cre lineages was required for normal SLV development. *Adamts5* mRNA (**A**,**B**, green, orange), endothelium (**A**,**B**, red, Pecam mRNA) in E12.5 OFT (**A**,**B**). Green arrowheads—overlap of *Pecam* and *Adamts5* mRNA. VCAN mRNA (**C**,**D**, green) in cusp mesenchymal cells. Boxes in (**A**,**C**) magnified in (**B**,**D**), respectively. *Adamts5^f/f^* (control, PV, **E**,**I**,**M**), ZP3-Cre global deletion (**F**,**J**,**N**). *Adamts5* Tie2-Cre lineage deletion (**G**,**H**,**K**,**L**,**O**,**P**). Cusp width quantified (**Q**,**R**) each symbol represents data from a single mouse. (**Q**): yellow circle—control PV-L; yellow square—ZP3-Cre global deletion PV-L; yellow triangle—Tie2-Cre PV-L; blue circle— control PV-R; blue square—ZP3-Cre global deletion PV-R; blue triangle—Tie2-Cre PV-R; red circle—control PV-An; red square—ZP3-Cre global deletion PV-An; red triangle—Tie2-Cre PV-An. Graph R: yellow circle—control PV-L; yellow diamond—Tnnt2-Cre deletion PV-L; blue circle—control PV-R; blue diamond—Tnnt2-Cre PV-R; red circle—control PV-An; red diamond—Tnnt2-Cre PV-An. * *p* < 0.05, ** *p* < 0.01, *** *p* < 0.001, **** *p* < 0.0001, from non-parametric tests, ns—not significant. Bar in (**A**) = 50 μm.

*Adamts5* mRNA is expressed in the endocardium of the prevalvular cusps (Figure 6A,B, green, orange) [7,25], while VCAN is primarily expressed by the VICs and myocardium at E12.5 (Figure 6C,D, green), indicating that ADAMTS5-mediated VCAN cleavage may facilitate inter-lineage communication in the remodeling valve cusps. To determine if expression of *Adamts5* by the endothelial cells and their mesenchymal derivatives (EndoMT) are required for normal semilunar valve development, the *Adamts5* floxed allele [26,27] was used in combination with Tie2-Cre (*Tie2^Cre^;Adamts5^f/f^*). Since this was the first use of the *Adamts5* floxed allele in cardiovascular biology, the germline global deletion (*ZP3^Cre^;Adamts5^f/f^*) was generated to ensure SLV phenotypes were similar to the global *Adamts5^−/−^* knockout mice used previously [7,25]. The global deletion of *Adamts5^f/f^* floxed allele, *ZP3^Cre^;Adamts5^f/f^,* (n = 7) resulted in significantly enlarged PV cusps at E17.5, a timepoint just prior to birth when *Adamts5^+/+^* semilunar valves have a sculpted morphology (Figure 6F,J,N,Q—squares). The E17.5 *Tie2^Cre^;Adamts5^f/f^* (n = 7) L-PV, R-PV, and An-PV cusps were significantly increased in width compared to controls (*Adamts5^f/f^*) (n = 14) (Figure 6G,H,K,L,O–Q—triangles). The An-PV cusp of the *Tie2^Cre^;Adamts5^f/f^* was significantly smaller than the global ZP3 deletion, suggesting a lineage other than Tie2 may contribute to the intercalated cushion-derived An-PV cusp. The AV E17.5 phenotype of the floxed global (*ZP3^Cre^;Adamts5^f/f^)* and EndoMT (Tie2^Cre^) lineage specific *Adamts5* deletions were also evaluated (Supplemental Figure S3).

#### 3.6.2. ADAMTS5 Was Required in the Myocardial Lineage for Normal SLV Formation

Since a majority of the An-PV cusp comprises the Tnnt2^Cre^ myocardial lineage, [21,22] the myocardial Cre, *Tnnt2^Cre^;Adamts5^f/f^* was used to remove expression of *Adamts5* from myocardial cells. The *Tnnt2^Cre^Adamts5^f/f^* hearts (n = 6) exhibited significantly larger An-PV and L-PV cusps than control mice (n = 14) (Figure 6R).

#### 3.6.3. ADAMTS5 Expression in the EndoMT Lineage Was Required to Form the Narrow Hinge Regions of the SLV Cusps

**Figure 7:**
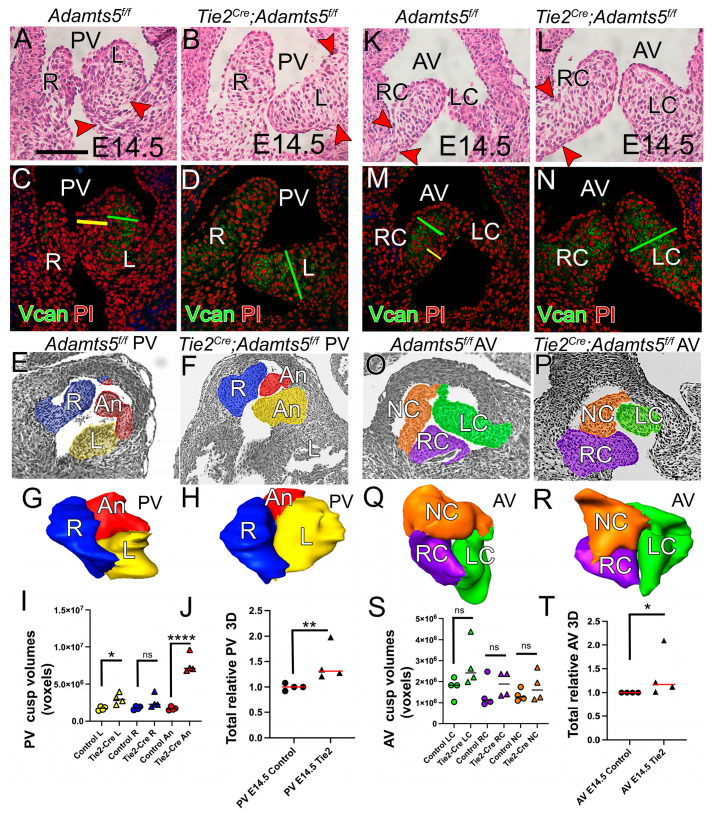
In E14.5 AV and PV of Tie2-Cre recombined *Adamts5^f/f^*, VCAN organization and morphology was disrupted. Sections of PV (**A**–**F**) and AV (**K**–**P**) of control *Adamts5^f/f^*—(n = 4; **A**,**C**,**E**,**K**,**M**,**O**) and *Tie2^Cre^;Adamts5^f/f^* (n = 4; **B**,**D**,**F**,**L**,**N**,**P**) valves. Red arrowheads-hinge regions of the E14.5 control *Adamts5^f/f^* (**A**,**K**) and *Tie2;Adamts5^f/f^* (**B**,**J**). Green lines—VCAN localization in control cusps (**C**,**M**) and *Tie2;Adamts5^f/f^* (**D**,**N**); yellow lines—compacted cells void of VCAN in controls (**C**,**M**). Amira™ Orthoslice with color identification of valve cusps (**E**,**F**,**O**,**P**); yellow—PV-L, blue—PV-R, red—PV-An, green—AV-LC, purple—AV-RC, orange—AV-NC. Three-dimensional reconstructions of the PV (**G**,**H**) and AV (**Q**,**R**). Graphs (**I**,**J**,**S**,**T**); each symbol represents a single mouse. Circles represent control *Adamts5^f/f^*, triangles indicate *Tie2^Cre^;Adamts5^f/f^*. Color of shape in graph corresponds to cusp denoted above. * *p* < 0.05, ** *p* < 0.01, **** *p* < 0.0001, * in (**I**), and (**S**) from parametric test, data from (**J**) and (**T**) non-parametric test, ns—not significant. Bar in 50 μm applies to (**B**–**R**).

A key morphological transition in cusp sculpting is the narrowing of the hinge region as VCAN is reduced and collagen I is assembled. At E14.5 the valve hinge regions narrow where the cusps anchor (Figure 7A,K, red arrowheads), but the corresponding *Tie2^Cre^;Adamts5^f/f^* hinge regions were wide and devoid of sculpting (Figure 7B,L, red arrowheads). In E14.5 control AV and PV (n = 4) the VICs adjacent to the endothelium (Figure 7C,M, yellow bars) were compacted, but in the *Tie2^Cre^;Adamts5^f/f^* cusps (n = 4) the compacted cell layer was not present (Figure 7D,N). Notably, loss of cell compaction in the *Tie2^Cre^;Adamts5^f/f^* cusps coincided with an expansion of VCAN localization (Figure 7D,N, green bars) compared to controls (Figure 7C,M, green bars). In addition, intact VCAN becomes restricted to the spongiosa layer, while cleaved VCAN is localized to the fibrosa layer with collagen and elastin. Collectively, these ECM changes are referred to as the initial stages of ECM stratification. These data indicated that expression of *Adamts5* in the EndoMT lineage may be critical to establish the early ECM stratification in murine SLV.

## 4. Discussion

### 4.1. OFT Lineages Exhibit Changes in Patterning Due to Loss of ADAMTS5

Factors that coordinate lineage specific cell behaviors are not well understood, but a critical role for VCAN cleavage may be emerging. In the developing OFT, cleaved VCAN overlaps with CNC localization, while intact VCAN colocalizes with the EndoMT lineage [21]. In other contexts, VCAN contributes to impenetrable borders along neural crest migratory routes, confining the motile neural crest cells and facilitating compaction in vivo [30,31,32]. The expression of ADAMTS5 by the endocardium may define an important ECM remodeling event that establishes the migratory route for CNC in the OFT [21,33]. This is consistent with the observation that disruption of VCAN cleavage in early OFT development resulted in reduced CNC lineage in the proximal OFT. The normal patterning of CNC in the prevalvular cushions was also disrupted by excess VCAN that correlated with AV and PV malformations. Lineage tracing studies also determined that the increased EndoMT lineage in the proximal region of the *Adamts5^−/−^* OFT did not compensate for the loss of CNC, suggesting that different mesenchymal cell lineages exhibit distinct behaviors in the remodeling OFT and are not interchangeable. Different roles between CNC and EndoMT are also evident in semilunar valves from mice with loss of the endothelial Brg1 chromatin remodeling complex. Endocardial *Brg1*-deficient mouse embryos develop thickened and disorganized semilunar valve cusps that become bicuspid and enlarged. The *Brg1*-deficient phenotype is due to defective EndoMT in the proximal OFT cushions. Although the missing cells are replaced by compensating CNC, these cells cannot fully pattern the specialized ECM associated with semilunar valve maturation [34]. Collectively, these studies indicate that each cell lineage is specified for distinct cell behaviors and interactions that may be dependent on ADAMTS5-mediated VCAN proteolytic cleavage in SLV development.

### 4.2. Initial Stratification of Cardiac Valve ECM Involves CNC Patterning and Vcan Cleavage

Establishing a highly organized ECM is required for normal SLV morphology. In this study, loss of endothelial expression of *Adamts5* disrupted CNC compaction and cleaved VCAN localization (E12.5) in abnormal semilunar valves. In other contexts [9,10], ADAMTS cleaved VCAN fragments termed ‘Versikine’ exhibit bioactivity distinct from the intact form, suggesting Versikine may also promote early valve ECM specification. By mid-gestation, *Adamts5* expression is localized to the ventricularis layer that contains elastin-rich ECM and intact VCAN is sequestered to the spongiosa layer. Collectively, these processes are involved in ECM stratification. There is also evidence that *Notch1* and *Notch2*, along with Notch ligand *Jagged1*, promote CNC patterning and when disrupted, result in valve malformations [35,36,37,38,39]. Like *Adamts5*, Notch1 activation is restricted to the valvular endothelial cells (VECs) on the ventricularis side of developing AV [40]. In contrast, well-spaced VICs colocalize with intact VCAN and the EndoMT lineage; the expansion of the spongiosa layer with the loss of CNC compaction near the endocardium in *Adamts5*-deficient hearts highlights the critical role of VCAN turnover to specialize ECM in SLV.

### 4.3. Proteolytic Cleavage of Fibronectin May Contribute to the Remodeling ECM in the Cardiac OFT

In addition to ADAMTS5 [7,41], ADAMTS19 [42], ADAMTS9 [41], and ADAMTS16 [43] have been shown to play a role in valve biology. While VCAN is a major ECM substrate of ADAMTS5 and ADAMTS9, ADAMTS16 exhibits catalytic activity on full-length fibronectin (FN) and generates fragments which are necessary for FN fiber assembly [44,45,46]. During cardiac development, FN promotes SHF progenitor cell proliferation, as well as CNC patterning, by binding to integrin receptors [47,48]. In mice, loss of *Adamts16* and the variant *Adamts16p.H357Q* result in BAVs with thickened cusps. The human variant *ADAMTS16p.H357Q* is associated with family members affected with a BAV [43]. Like *Adamts5*, expression of *Adamts16* is critical in the endothelial and myocardial lineage of the SHF [43]. Examination of *Adamts16 deficient* heart tissue revealed elevated FN levels. Since FN interacts directly with integrins, the excess FN in *Adamts16^+/−^* OFT may disrupt normal cell–matrix interactions. FN is a key ECM component of early valve formation and potentially another example of how loss of an ADAMTS proteolytic cleavage leads to clinically relevant AV malformations.

### 4.4. Reciprocal Interactions of ECM Cleavage Events and Mechanical Force May Be Required for Early ECM Organization

Since ECM composition is a direct reflection of the biomechanical forces that cells endure, deciphering the mechanosensing mechanisms that impart ECM specialization is an important, albeit challenging, area of valve biology. In fetal valve sculpting, the SLV of the *Adamts5^−/−^* mice are enlarged and malformed. At this stage there is dramatic remodeling that is spatiotemporally specific with respect to the outflow and inflow sides of the valve cusps [49,50]. ADAMTS5 is expressed on the inflow side [25] where Notch signaling is required [51] and activated by laminar shear stress [49,50] to control of proteoglycan content [40]. Appropriate levels of Notch signaling are also required for CNN migration in OFT development like *Adamts5*-deficient mice [50,52]. In addition, ADAMTS5-deficient valves exhibit a transient phenotypic correction that correlates with increases in additional ECM proteoglycanases and increased blood flow after birth [53]. The shear stress-responsive transcription factor Kruppel-like factor 2 (KLF2) is also expressed on the ventricularis side of the valves [54] similar to *Adamts5* [7,25]. KLF2 and KLF4 upregulate *Adamts5* expression in endocardial cells and reduction in hemodynamic forces confers matrix deposition and valve thickening similar to that observed in ADAMTS5-deficient valves. Loss of endocardial expression of *Adamts19* also perturbs shear stress signaling and leads to proteoglycan deposition in cardiac valves [28,35]. *Klf2* and *Adamts5* are upregulated in the *Adamts19^−/−^* valvular endocardium, indicating potential pathway interactions between proteoglycan cleavage and mechanosensing. A previous study suggested that FN dimers have multiple integrin-binding sites that can activate the Focal Adhesion kinase (FAK) signaling pathway [55]. The interaction of FN with FAK complexes, where integrins link to the cytoskeleton, are key sites for transmission of mechanical forces from the ECM. Collectively, these studies may indicate that involvement of ADAMTS proteases in mechanosensing pathways is required for ECM stratification during valve formation.

### 4.5. Limitations of the Study

The approach of this study was limited to the use of an animal model to recapitulate aspects of human valve disease. Given the dramatic size differences between the two, human AV tissue is likely to have different ECM profiles of proteoglycans compared to mice. However, the basic molecular mechanisms and pathways are generally recapitulated between mice and humans, making them an important pre-clinical model of disease. In future studies, examining additional ECM components, including fibronectin, that are altered due to proteolytic cleavage, would advance the understanding of the spatiotemporal ECM remodeling events that contribute to the forming of valve cusps.

## 5. Conclusion

Data in this study highlight the diversity of early mesenchymal cells in the OFT that are required to enable production and modification of the ECM landscape. Understanding the control of proteolytic cleavage of ECM components, including VCAN, may lead to effective pharmacological treatment of valve dysfunction and/or biomarkers that trace the progression of AV disease and aortopathies.

## Data Availability

The original contributions presented in this study are included in the article/Supplementary Material. Further inquiries can be directed to the corresponding author.

## Supplementary Materials

The following supporting information can be downloaded at: https://www.mdpi.com/article/10.3390/jcdd12090371/s1, Figure S1: Schematics of Lineage Tracing Strategy in *Adamts5^+/+^* and *Adamts5^−/−^* mice; Figure S2: Conditional inactivation of *Adamts5* using the floxed *Adamts5* allele and lineage specific promoters; Figure S3: 3D reconstructions revealed increased size and altered morphology of the ZP3-Cre and Tie2-Cre *Adamts5* floxed allele AV at E17.5.

## Abbreviations

The following abbreviations are used in this manuscript:

ADAMTS5	A disintegrin and metalloproteinase with thrombospondin motifs 5
AV	aortic valve
ECM	extracellular matrix
VCAN	versican
SLV	semilunar valves (aortic and pulmonary valves)
CNC	cardiac neural crest
EndoMT	endothelial to mesenchymal transformation
BAV	bicuspid aortic valve
FN	fibronectin
OFT	outflow tract
VIC	valvular interstitial cells
SHF	secondary heart field
H&E	hematoxylin and Eosin
EGFP	enhanced green fluorescent protein
