# Supplementary material for: ADAMTS5 Orchestrates Cell Lineage Specific Patterning and Extracellular Matrix Organization During Semilunar Valve Development
Source: J Cardiovasc Dev Dis. 2025 Sep 19;12(9):371. doi: 10.3390/jcdd12090371 (PMC12470463; doi:10.3390/jcdd12090371)
Supplement: Supplementary file 1 [file jcdd-12-00371-s001.zip › jcdd-3853901-supplementary.pdf]

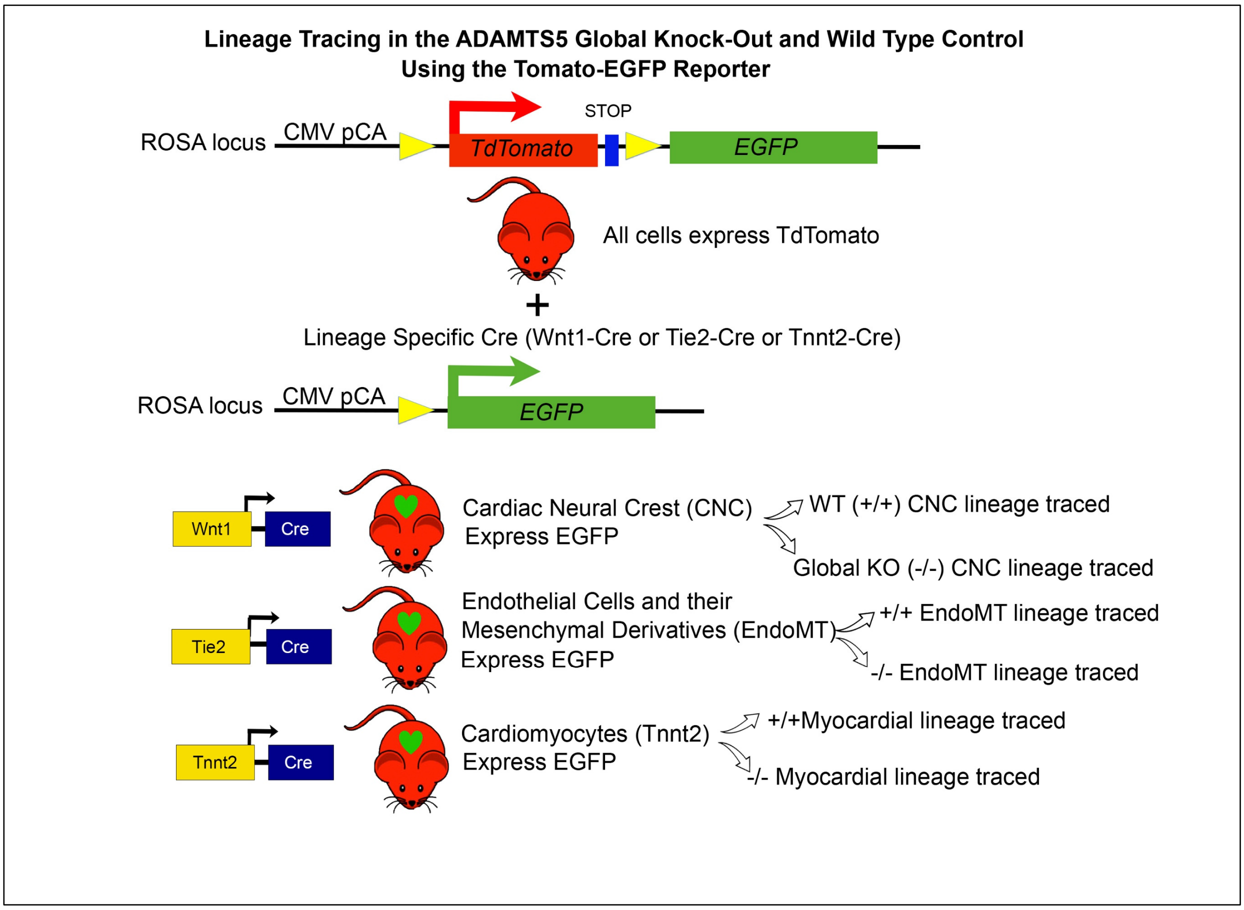

Supplemental Figure 1: Schematics of Lineage Tracing Strategy in *Adamts5*$^{+/+}$ and *Adamts5*$^{-/-}$ mice. To perform lineage tracing the Wnt1-Cre, Tie2-Cre or Tnnt2-Cre promoter with the Cre encoding transgene was used in combination with the tdTomato-EGFP reporter in the *Adamts5*$^{-/-}$ or control, *Adamts5*$^{+/+}$ mice. Without the Cre recombinase the reporter expresses TdTomato flourecence and the EGFP gene is not expressed. Upon Cre expression the tdTomato gene was excised in the Cre positive cells, which allows expression of EGFP i.e. effectively tracing either the Wnt1-Cre, Tie2-Cre or Tnnt2-Cre lineage.

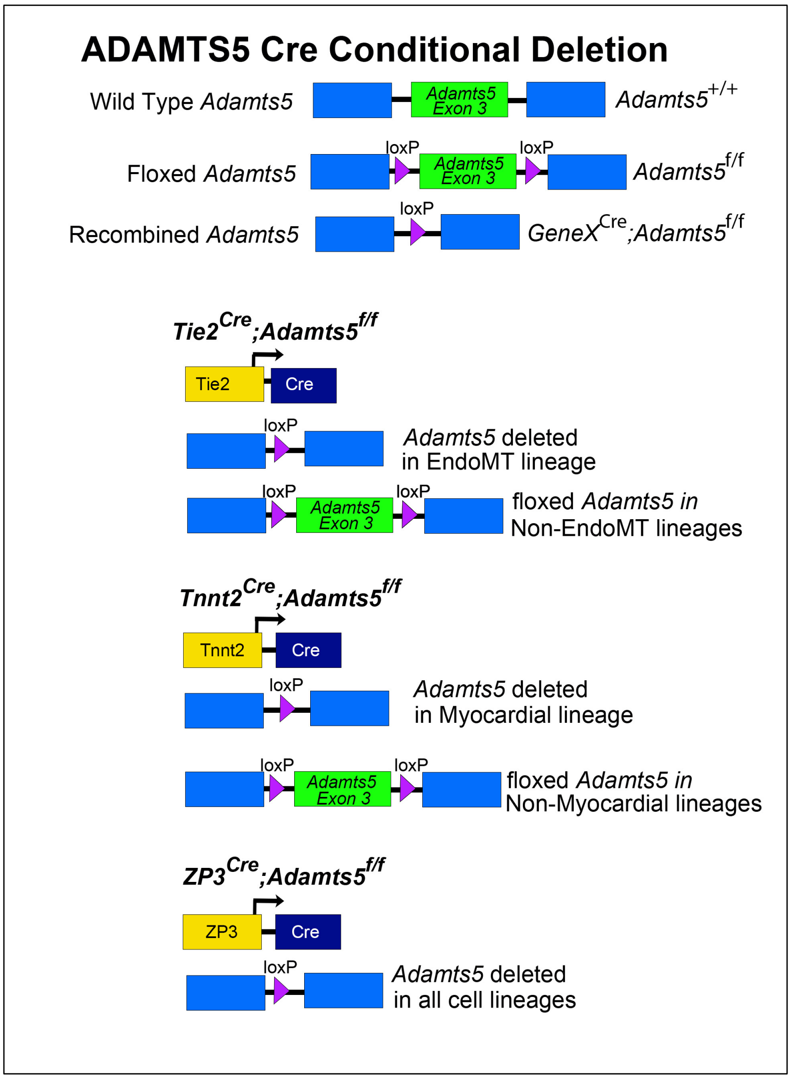

Supplemental Figure 2: Conditional inactivation of *Adamts5* using the floxed *Adamts5* allele and lineage specific promoters, Tie2, Tnnt2, or Zp3 (germline global deletion control) to conditionally inactivate *Adamts5* using the Cre recombinase. To determine the requirement of *Adamts5* mRNA expression in a specific lineage we utilized the *Adamts5* floxed allele designated (*Adamts5*$^{f/f}$) [26, 27]. In combination with a Cre transgene. Cre inactivation of *Adamts5*$^{fl/fl}$ removes exon 3 that encodes the proteoglycan cleavage domain These mice were used to investigate the contribution of *Adamts5* from Tie2-Cre or Tnnt2-Cre OFT lineages during development. The ZP3-Cre reporter was used as a positive control and to ensure the use of the germline deletion in the floxed allele was similar to the global knock-out used in the lineage tracing studies.

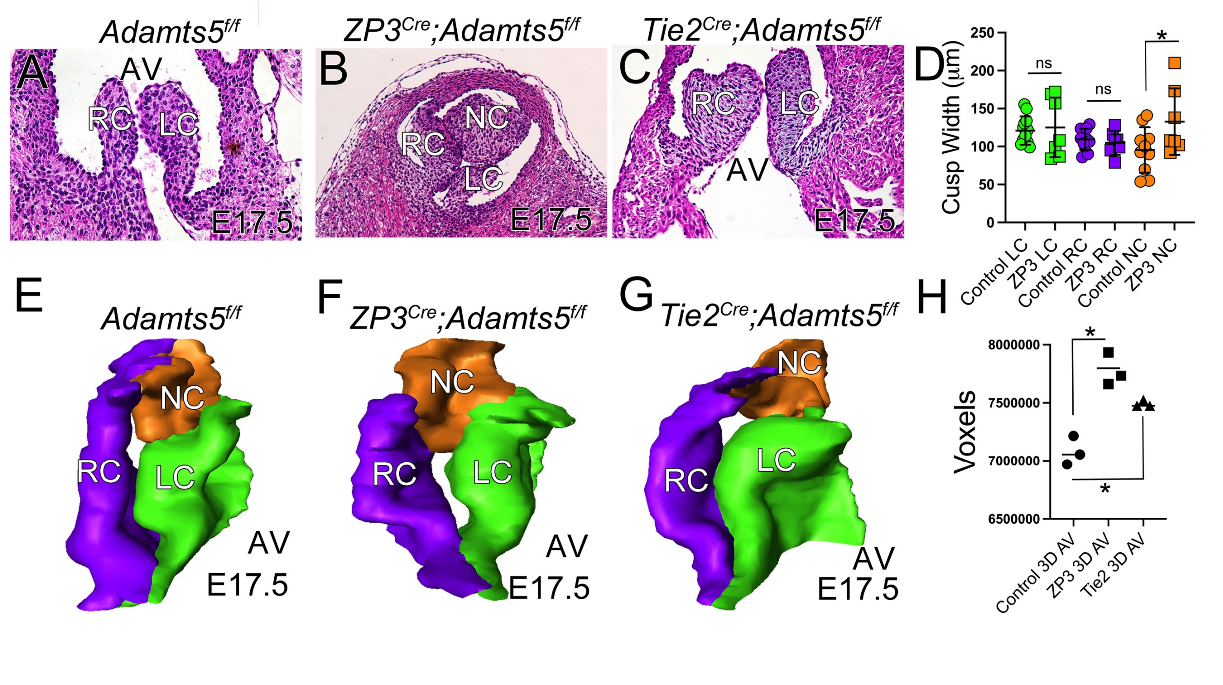

Supplemental Figure 3: 3D reconstructions revealed increased size and altered morphology of the ZP3-Cre and Tie2-Cre *Adamts5* floxed allele AV at E17.5. H&E images of sections through the AV of *Adamts5$^{f/f}$* (A), *ZP3^{Cre};Adamts5$^{f/f}$* (B) and *Tie2^{Cre};Adamts5$^{f/f}$* (C). Cusp width measured in histological sections and graphed in D: Green circles-control AV-LC; green squares-*ZP3^{Cre};Adamts5$^{f/f}$*, AV-LC; purple circles-control AV-RC; purple squares-*ZP3^{Cre};Adamts5$^{f/f}$* AV-RC; orange circles-control AV-NC; orange squares- *ZP3^{Cre};Adamts5$^{f/f}$* AV-NC. 3D reconstructions shown in E, F, and G. Total AV volume quantified in H; black circles-control 3D AV, black squares- *ZP3^{Cre};Adamts5$^{f/f}$* 3D AV, black triangles-*Tie2^{Cre};Adamts5$^{f/f}$* 3D AV). *$P<0.05$, ns-not significant. Bar in A=50μm applies to B, C, E-G.
